# A Genetic Variant in the IL-17 Promoter Is Functionally Associated with Acute Graft-Versus-Host Disease after Unrelated Bone Marrow Transplantation

**DOI:** 10.1371/journal.pone.0026229

**Published:** 2011-10-20

**Authors:** J. Luis Espinoza, Akiyoshi Takami, Katsuya Nakata, Makoto Onizuka, Takakazu Kawase, Hideki Akiyama, Koichi Miyamura, Yasuo Morishima, Takahiro Fukuda, Yoshihisa Kodera, Shinji Nakao, for the Japan Marrow Donor Program

**Affiliations:** 1 Department of Hematology and Oncology, Kanazawa University Hospital, Kanazawa, Japan; 2 Department of Hematology and Oncology, Tokai University School of Medicine, Isehara, Japan; 3 Division of Epidemiology and Prevention, Aichi Cancer Research Center, Nagoya, Japan; 4 Hematology Division, Tokyo Metropolitan Cancer and Infectious Diseases Center, Komagome Hospital, Tokyo, Japan; 5 Department of Hematology, Japanese Red Cross Nagoya First Hospital, Nagoya, Japan; 6 Hematopoietic Stem Cell Transplantation Unit, National Cancer Center Hospital, Tokyo, Japan; 7 Department of Promotion for Blood and Marrow Transplantation, Aichi Medical University, Nagoya, Japan; Instituto Nacional de Câncer, Brazil

## Abstract

Interleukin IL-17 is a proinflammatory cytokine that has been implicated in the pathogenesis of various autoimmune diseases. The single nucleotide polymorphism (SNP), rs2275913, in the promoter region of the IL-17 gene is associated with susceptibility to ulcerative colitis. When we examined the impact of rs2275913 in a cohort consisting of 438 pairs of patients and their unrelated donors transplanted through the Japan Marrow Donor Program, the donor IL-17 197A allele was found to be associated with a higher risk of acute graft-versus-host disease (GVHD; hazard ratio [HR], 1.46; 95% confidence interval [CI], 1.00 to 2.13; *P* = 0.05). Next, we investigated the functional relevance of the rs2275913 SNP. *In vitro* stimulated T cells from healthy individuals possessing the 197A allele produced significantly more IL-17 than those without the 197A allele. In a gene reporter assay, the 197A allele construct induced higher luciferase activity than the 197G allele, and the difference was higher in the presence of T cell receptor activation and was abrogated by cyclosporine treatment. Moreover, the 197A allele displayed a higher affinity for the nuclear factor activated T cells (NFAT), a critical transcription factor involved in IL-17 regulation. These findings substantiate the functional relevance of the rs2275913 polymorphism and indicate that the higher IL-17 secretion by individuals with the 197A allele likely accounts for their increased risk for acute GVHD and certain autoimmune diseases.

## Introduction

Interleukin 17 (IL-17), also known as IL-17A, plays an important role in tissue inflammation, and is involved in the pathophysiology of autoimmune diseases and organ allograft rejection [1], [2], [3], [4], [5], [6], [7], [8], [9], [10], [11]. Moreover, several reports have shown that Th17 cells and IL-17 have a significant impact on the development of acute graft-versus-host disease (GVHD) in mouse models [12], [13], [14], [15], [16], [17]. The 197A allele, which is the result of a single nucleotide polymorphism (SNP) rs2275913 (G197A) in the promoter region of the IL-17 gene, has been reported to be associated with the susceptibility to rheumatoid arthritis [18] and ulcerative colitis [19]. In our previous study, we demonstrated that the 197A allele was also implicated in the development of acute GVHD in patients who underwent unrelated myeloablative bone marrow transplantation (BMT) [20]. In the present study, we extended this investigation to a validation cohort of patients who received an unrelated BMT, including patients who underwent reduced intensity transplantation.

Interestingly, the rs2275913 SNP is located within a binding motif for the nuclear factor activated T cells (NFAT), which is a critical regulator of the IL-17 promoter [21]. Therefore, it is conceivable that the rs2275913 SNP exerts an effect on the transcriptional regulation of IL-17. The present study shows that allele 197A correlates with more efficient IL-17 secretion, and that this resulted from its higher affinity for NFAT.

## Materials and Methods

### Patients

IL-17 genotyping was performed on 438 recipients with hematological malignancies and their unrelated donors who underwent BMT through the Japan Marrow Donor Program (JMDP) with T-cell-replete marrow from HLA-A, -B, -C, -DRB1, -DQB1, and -DPB1 allele-matched donors between January 1993 and December 2007. The HLA genotypes of patients and donors were determined by the Luminex microbead method as described previously (Luminex 100 System; Luminex, Austin, TX) [22], [23]. Although the Luminex microbead method does not provide unambiguous HLA 4-digit typing for all genotypes, the JMDP has confirmed that this method can identify all HLA alleles with >0.1% frequency among the Japanese population [24].

None of the present patients had a history of any prior transplantation. The final clinical survey of these patients was completed by November 1, 2008. The diagnoses were acute myeloid leukemia (AML) in 149 (34%), acute lymphoblastic leukemia (ALL) in 109 (25%), myelodysplastic syndrome (MDS) in 78 (18%), malignant lymphoma (ML) in 55 (15%), chronic myeloid leukemia in 42 (10%), and multiple myeloma (MM) in 5 (1%; **Tables 1** and **2**). The recipients were defined as having standard risk disease if they had AML or ALL in the first complete remission, ML in any complete remission, CML in any chronic phase, or MDS. All others were designated as having high-risk disease. The myeloid malignancies include AML, MDS and CML, and the lymphoid malignancies included ALL, ML and MM. Cyclosporine- or tacrolimus-based regimens were used in all patients for GVHD prophylaxis, and anti-T cell therapy, such as anti-thymocyte globulin and *ex vivo* T cell depletion were not in any of the patients. All patients and donors gave their written informed consent at the time of transplantation to participate in molecular studies of this nature according to the declaration of Helsinki. This project was approved by the Institutional Review Board of Kanazawa University Graduate School of Medicine and the JMDP.

**Table 1:** The donor and recipient characteristics (first part).

Variable	No.	Ratio
No. of cases	438	
Recipient age, years		
Median	39	
Range	1–70	
Donor age, years		
Median	35	
Range	20–57	
Year of transplant		
Median	2003	
Range	1993–2007	
Recipient IL-17 genotype		
G/G	180	41%
A/G	200	46%
A/A	58	13%
Donor IL-17 genotype		
G/G	166	38%
A/G	200	46%
A/A	66	15%
Recipient sex		
Male	281	64%
Female	157	36%
Donor sex		
Male	296	68%
Female	142	32%
Donor/recipient sex		
Sex matched	299	68%
Female/male	62	14%
Male/female	77	18%

**Table 2:** Donor and recipient characteristics (second part).

Variable	No.	Ratio
Disease		
Acute myeloid leukemia	149	34%
Acute lymphoblastic leukemia	109	25%
Myelodysplastic syndrome	78	18%
Malignant lymphoma	55	13%
Chronic myeloid leukemia	42	10%
Multiple myeloma	5	1%
Disease stage		
Standard risk	178	41%
High risk	260	59%
ABO matching		
Major or/and minor mismatch	160	37%
Major mismatch	91	21%
Minor mismatch	86	20%
Bidirectional	17	4%
Missing	8	2%
Conditioning regimen		
Myeloablative	325	74%
Reduced intensity	113	26%
With total body irradiation	333	76%
Pretransplant CMV serostatus		
CMV positive recipient	324	74%
Missing	40	9%
GVHD prophylaxis		
With cyclosporine	190	43%
With tacrolimus	248	57%
TNC, ×10^8^ per kg		
Median	4.6	
Range	0.1–316.8	

Abbreviations: TNC: total nucleated cell count harvested.

### IL-17 genotyping

Genotyping of IL-17 was performed using the TaqMan-Allelic discrimination method with the Assay ID C__15879983_10 (Applied Biosystems) as described in a previous report [20].

### Cells and reagents

Primers and oligonucleotides were obtained from Hokkaido Science Systems (Sapporo, Japan). The GST-NFATc1 construct [25] was a generous gift from Dr Shoichiro Miyatake. An NFATc binding consensus oligonucleotide (sc-2577) was purchased from Santa Cruz Biotechnology (Santa Cruz, California).

### Cell preparation, cell culture and measurement of IL-17

Heparinized blood samples were collected from 54 healthy Japanese volunteers. The ages of the subjects (30 males and 24 females) ranged from 20 and 56 years (median, 32 years). Peripheral blood mononuclear cells (PBMCs) were isolated using a Ficoll-Hypaque gradient (Pharmacia Biotech, Uppsala, Sweden) and were induced to secrete IL-17 by culturing the PBMCs (10^6^/well) in 24 well plates for 48 hours in RPMI 1640 supplemented with 10% fetal bovine serum in the presence or absence of 5 µg/ml phytohemagglutinin (PHA; Sigma) at 37°C in 5% CO_2_. In some experiments, PBMCs (10^6^ cells/well) were seeded in 48 well plates coated with anti-CD3 (2 µg/ml) and anti-CD28 (1 µg/ml) monoclonal antibodies (Miltenyi Biotec, Gladbach, Germany) to activate T cells selectively, and then were cultured for 48 hrs. The concentrations of IL-17 in collected supernatants were measured by an enzyme-linked immunosorbent assay (ELISA; Mabtech, Nacka Strand, Sweden). For some functional assays, PBMCs (6×10^6^ cells/well) were cultured in six well plates for 72 hrs in the presence of 5 µg /ml of PHA and 100 U/ml of IL-2, and are hereafter designated as PHA-PBMCs.

### Quantitative RT- PCR

RNA was extracted from resting or PHA-activated PBMCs using the high pure RNA isolation kit (Roche). Reverse transcription was carried out with the PrimeScript RT reagent/gDNA eraser kit (Catalog RR047A, Takara). Quantitative real time PCR was performed in a StepOne Plus PCR system (Applies Biosystems) using the SYBR premix ExTaq perfect Real Time (Catalog RR041A, Takara) with the IL-17 primers described previously [26] and a set of primers for human GAPDH (Takara). The relative IL-17 mRNA levels normalized to GAPDH were calculated by the ΔΔCT method using the relative expression function included in the StepOne v2.2 software program. The specificity of the PCR products was monitored by a melting curves analysis.

### Luciferase assay

The promoter region of the IL-17 gene was amplified from the genomic DNA of individuals homozygous for the rs2275913 SNP (A197A or G197G) by polymerase chain reaction (PCR) with forward 5′-ACGCGTGGATCTCAGGACAAACAGGTTC-3′ and reverse 5′-AAGCTTGACTCACCACCAATGAGGTCTT-3′ primers as described previously [21]. The resultant fragments IL-17/197A or IL-17/197G were subcloned into the pGL3-enhancer vector at the MluI and HindIII sites (Promega, Madison, WI) to generate pGL3-197A-enhancer or pGL3-197G-enhancer constructs. The fragments were inserted with the same orientation, and their nucleotide sequences were confirmed by DNA sequencing. Equimolar amounts of the following reporter plasmids: pGL3-enhancer, pGL3-197A-enhancer and pGL3-197G enhancer designated thereafter as pGL3-Luc, IL-17/A-Luc IL-17/G-Luc respectively, were transfected into PHA-PBMCs using the Exfect transfection reagent following the manufacturer instructions (Takara Bio, Japan). To control for differences in the transfection efficiency, cells were cotransfected with a renilla reporter plasmid, pRL-TK. In some experiments, the cells were treated with anti-CD3 and anti-CD28 mAbs or with Cyclosporine A (CsA) 24 hours after the transfection, and were cultured for other 24 hours. The activity of both luciferase and renilla in the transfected cells was measured with the Dual Luciferase Reporter Assay System (Promega).

### Electrophoresis motility shift assay (EMSA)

Double stranded IL-17 probes, including those harboring G197A, were generated by annealing the following oligonucleotides to their complementary oligonucleotides: CAT TTT CCT TCA GAA G**A**A GAG ATT CTT CTA (197A allele) and CAT TTT CCT TCA GAA G**G**A GAG ATT CTT CTA (197G allele). These oligomers encompass nucleotides −180 to −210 upstream of the transcriptional start site, based on data in the human genomic DNA Gene bank accession number AY460616.1. Before annealing, both complementary oligonucleotides were separately biotin-labeled at their 3′ ends, using the 3′ end DNA labeling kit (Thermo Fisher Scientific, Suwanee, USA) following the manufacturer's recommendations. Nuclear extracts from PHA-PBMCs were prepared using a nuclear extraction kit (Thermo Fisher Scientific). The DNA/protein binding assay was performed with 10 µg of nuclear extracts using the Light Shift Chemiluminescent EMSA kit (Thermo Fisher Scientific) according to the manufacturer's recommendations with minor modifications as follows: In the DNA/NFAT recombinant protein assay 0.5% bovine serum albumin was included in the binding reaction and purified GST-NFAT-recombinant proteins were desalted using Zeba spin desalting columns (Pierce). The DNA/protein complexes were detected by streptavidin peroxidase and visualized in a Luminescent Image Analyzer LAS-4000 (Fujifilm, Tokyo, Japan).

### *Data management and statistical analysis*

The data were collected by the JMDP using a standardized report form. Follow-up reports were submitted at 100 days, 1 year and annually after transplantation. The pre-transplant cytomegalovirus (CMV) serostatus was routinely tested for only patients, but not the donors. Engraftment was confirmed by an absolute neutrophil count of more than 0.5×10^9^/L for at least 3 consecutive days. After collecting the data, acute and chronic GVHD were diagnosed and graded based on the classically defined criteria [27], [28], namely, acute GVHD develops within the first 100 days post-transplant while the manifestation of GVHD occurring after day 100 is classified as chronic GVHD. Data using the updated criteria for assessment of GVHD [29], [30] were not available in our cohort. The overall survival (OS) was defined as the number of days from transplantation to death from any cause. Disease relapse was defined as the number of days from transplantation to disease relapse. Transplant-related mortality (TRM) was defined as death without relapse. Any patients who were alive at the last-follow-up date were censored. The data about the causative microbes of infections and postmortem changes in the cause of death, as well as the data on supportive care, including prophylaxis for infections and therapy for GVHD, which were given on an institutional basis, were not available for this cohort.

The analysis was performed using the Excel 2007 software program (Microsoft Corp, Redmond, WA, USA) and modified R (The R Foundation for Statistical Computing, Perugia, Italy) software programs [31], [32], as described in a previous report [33], [34]. The probability of OS was calculated using the Kaplan-Meier method and compared using the log-rank test. The probabilities of TRM, disease relapse, acute GVHD, chronic GVHD, and engraftment were compared using the Grey test [35] and analyzed using a cumulative incidence analysis [31], while considering relapse, death without disease relapse, death without acute GVHD, death without chronic GVHD, and death without engraftment as respective competing risks. The variables included the recipient age at the time of transplantation, sex, pretransplant CMV serostatus, disease characteristics (disease type, disease lineage and disease risk at transplantation), donor characteristics (age, sex, sex compatibility, and ABO compatibility), transplant characteristics (conventional or reduced-intensity conditioning [36], total body irradiation-containing regimen, tacrolimus versus cyclosporine, and total nucleated cell count harvested per recipient weight [TNC]), and the year of transplantation. The median was used as the cutoff point for continuous variables. The chi-square test and the Mann-Whitney U test were used to compare the two groups. The Hardy-Weinberg equilibrium for the IL-17 gene polymorphism was determined using the Haploview software program [37].

Multivariate Cox models were used to evaluate the hazard ratio associated with the IL-17 polymorphism. The covariates found to be P≤0.10 according to univariate analyses were used to adjust the hazard ratio.

For both the univariate and multivariate analyses, the P values were two sided, and the outcomes were considered to be significant for P≤0.05.

## Results

### Transplant outcome according to the IL-17 genotype

The genotype frequencies of 197G/G, 197A/G and 197A/A were 41%, 46% and 13% in recipients, and 38%, 46% and 15% in donors. These were similar to previous reports [18], [38], and were in accord with the Hardy-Weinberg equilibrium (*P* = 0.88).

**Figure 1:**
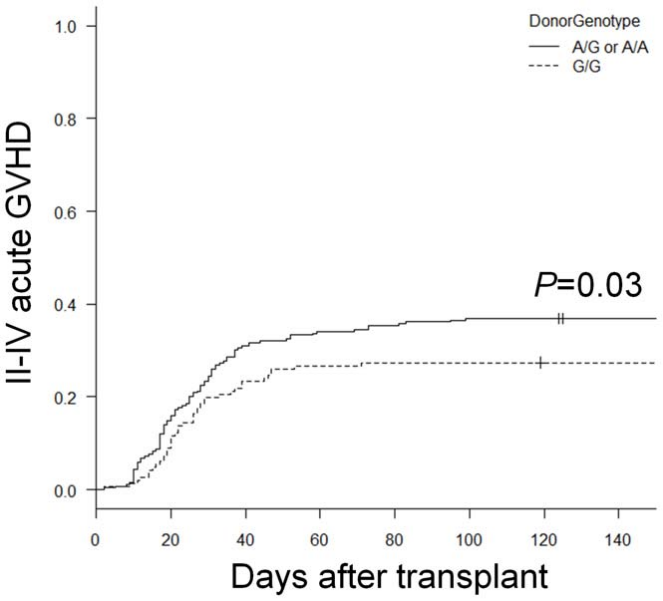
The estimated cumulative incidence curve of grades II–IV acute GVHD according to the donor IL-17 genotype. The solid line represents the donor 197A/G or A/A genotype, and the dashed line represents the donor 197G/G genotype.

The transplant outcomes according to the IL-17 genotype are summarized in **Table 3**. The presence of the 197A allele in the 197A/G or 197A/A genotype in the donor was associated with a significantly higher incidence of grades II to IV acute GVHD (38% vs. 27%, *P* = 0.03; **Fig. 1**), while no significant differences between the 197A/G genotype and the 197A/A genotype in the recipient were seen in the incidence of grades II to IV acute GVHD (38% vs. 36%, *P* = 0.78). The acute GVHD-related mortality did not differ between the donor 197A/G or 197A/A genotypes and the donor 197G/G genotype (2% vs. 2%, *P* = 0.83).

**Table 3:** The results of the univariate analysis of the association of IL-17 polymorphisms with the clinical outcomes after transplantation.

Variable	No.	5-year OS	*P*	5-year TRM	*P*	5-year relapse	*P*	II–IV acute GVHD	*P*	Chronic GVHD	*P*
Recipient IL-17 genotype											
G/G	180	41%		29%		37%		35%		41%	
A/G or A/A	258	50%	0.59	28%	0.48	30%	0.10	30%	0.21	40%	0.78
Donor IL-17 genotype											
G/G	166	50%		29%		31%		27%		37%	
A/G or A/A	272	43%	0.22	28%	0.71	33%	0.77	38%	0.03	42%	0.18

All of the factors found to be significant in the univariate analyses were included in the model. The 197A/G or 197A/A genotype in donors remained statistically significant in the multivariate analyses for the development of grades II to IV acute GVHD (**Table 4**). The 197A/G or 197A/A genotype in the donor resulted in a higher incidence of grades II to IV acute GVHD (hazard ratio [HR], 1.46; 95% confidence interval [CI], 1.00 to 2.13; *P* = 0.05) even when adjusted for the other factors in the models. The IL-17 genotype showed no significant effects on the OS, TRM or relapse (**Table 5**).

**Table 4:** The results of the multivariate analysis of the association of IL-17 polymorphisms with the GVHD after transplantation.

	II–IV acute GVHD			Chronic GVHD		
Variable	Adjusted HR	95% CI	P	Adjusted HR	95% CI	P
Recipient IL-17 genotype, A/G or A/A	0.80	0.56–1.13	0.20	1.32	0.86–1.03	0.21
Donor IL-17 genotype, A/G or A/A	1.46	1.00–2.13	0.05	1.08	0.70–1.67	0.72

**Table 5:** The results of the multivariate analysis of the association of IL-17 polymorphisms with the clinical outcomes after transplantation.

	OS			TRM			Relapse		
Variable	Adjusted HR	95% CI	P	Adjusted HR	95% CI	P	Adjusted HR	95% CI	P
Recipient IL-17 genotype, A/G or A/A	1.01	0.71–1.42	0.97	1.43	0.84–2.41	0.87	0.75	0.49–1.16	0.19
Donor IL-17 genotype, A/G or A/A	1.29	0.90–1.84	0.16	1.31	0.75–2.31	0.34	1.24	0.79–1.93	0.35

### The impact of the rs2275913 SNP on the secretion of IL-17

**Figure 2:**
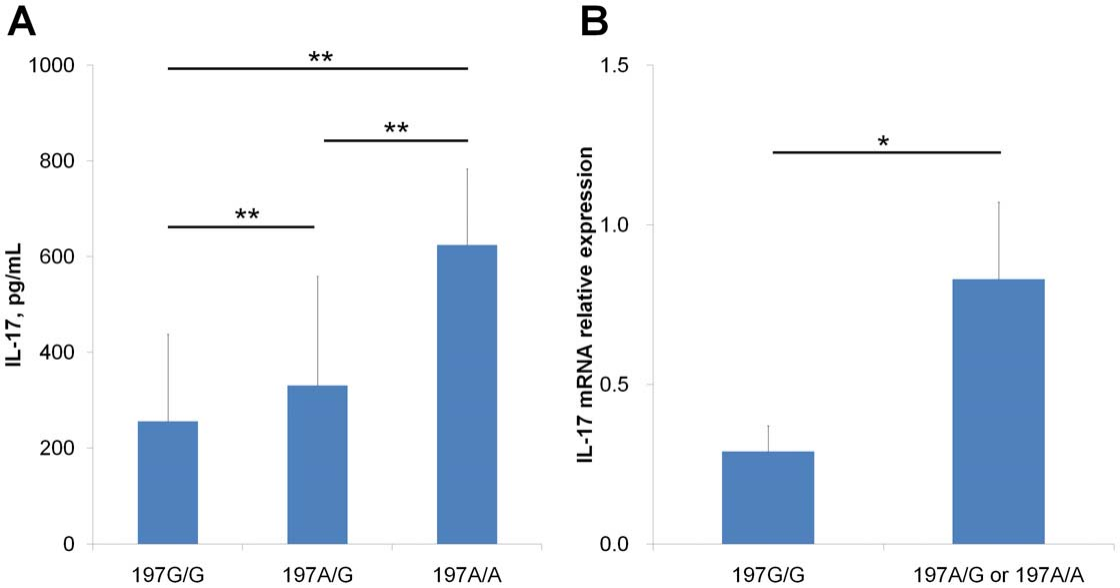
The influence of the IL-17 SNP on IL-17 secretion by *in vitro* stimulated PBMCs. (A) PBMCs from healthy individuals were stimulated for 48 hours in the presence of PHA or in 48 well plates coated with anti-CD3 and anti-CD28 mAbs. The values are expressed as the means +/− SD. (B) PBMCs from healthy donors (197A allele positive, n = 12 and 197A allele negative, n = 10) were cultured for 24 hrs in the presence of PHA. Total RNA was extracted, and the IL-17 mRNA levels were determined by quantitative RT-PCR and normalized to GAPDH. The data are the means +/−SD of triplicate measurements in each donor. *indicates *P*<0.05 and **P<0.01.

To substantiate the biological significance of the rs2275913 SNP, we first examined whether the different genotypes correlated with IL-17 secretion. PBMCs from a total of 54 healthy individuals (197G/G in 24, 197A/G in 24 and 197A/A in 6) were stimulated *in vitro* with PHA and the levels of secreted IL-17 were determined by ELISA. As shown in **Fig. 2A**, the 197A allele positive (197A/G or 197A/A genotype) PBMCs secreted significantly higher levels of IL-17 than the 197A allele negative cells (197G/G genotype). Similar results were obtained when T cells were selectively stimulated with anti-CD3 and anti-CD28 mAbs (data not shown). The quantitative RT-PCR analysis showed that PHA-stimulated PBMCs from donors harboring the 197A allele had a significantly higher IL-17 mRNA level than those from 197A allele negative donors (**Fig. 2B**). Of note, the IL-17 mRNA levels in unstimulated cells were very low, irrespective of 197A allele positivity, and resulted in no differences between the two groups (data not shown). Together, these results suggested that the sequence variant rs2275913 influences the response of the IL-17 gene promoter to factors released in response to T cell activation, thus leading to a differential IL-17 production.

### Functional relevance of the rs227513 SNP in the IL-17 gene promoter region

**Figure 3:**
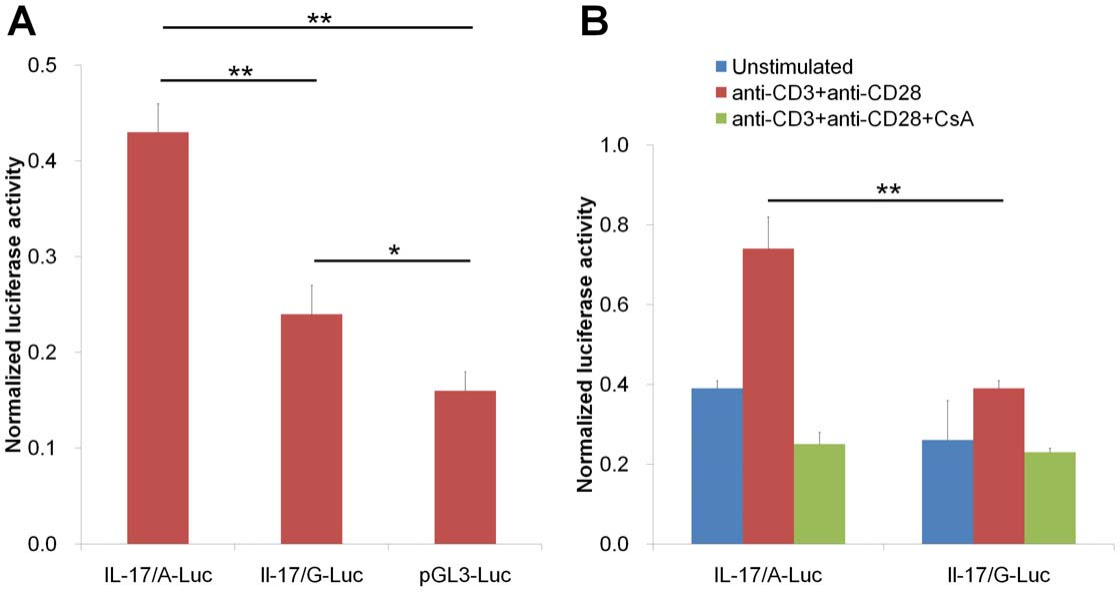
The modulation of the reporter gene expression by the rs2275913 SNP. (A) PHA-PBMCs were transfected with a luciferase expression vector alone (pGL3-Luc) or with a luciferase expression vector containing fragments of the IL-17 promoter with the 197A or 197G alleles (IL-17/A-Luc and IL-17/G-Luc). The transfected cells were cultured for 48 hr, and firefly luciferase activities were measured and normalized to Renilla luciferase. (B) The PHA-PBMCs were transfected as described above. Twenty four hours after transfection, the cells were treated with anti-CD3 and anti-CD28 mAbs or with CsA, and cultured for other 24 hr. The firefly luciferase activities were measured and normalized to Renilla luciferase. The values represent the normalized levels +/− S.E.M. from five independent experiments. *indicates *P*<0.05 and **P<0.01.

To address the functional significance of the rs2275913 SNP, reporter gene constructs containing the 197A and 197G alleles were prepared and used to transfect PHA-PBMCs. The results revealed that the insertion of the IL-17 promoter fragment consistently resulted in an augmentation of the luciferase activity compared with the construct without the fragments, however, the cells transfected with the IL-17/A-Luc construct had significantly higher luciferase activity than cells transfected with the IL-17/G-Luc construct (**Fig. 3A**). The differences in luciferase expression induced by these two constructs were more evident when the transfected cells were treated with anti-CD3 and anti-CD28 mAbs (**Fig. 3B**). Notably, treatment of the transfected cells with CsA abrogated the differences in luciferase activity induced by the two alleles, thus suggesting that the effects of the rs2275913 SNP on the regulation of the IL-17 promoter function are dependent on T cell activation.

### The 197A allele has a stronger interaction with NFAT than the 197G allele

**Figure 4:**
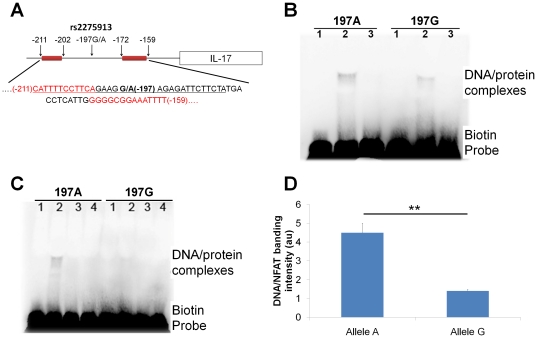
The rs2275913 SNP results in differential binding of NFAT. (A) The location of the rs2275913 SNP within the promoter region of the IL-17 gene. The underlined sequence corresponds to the oligomers used in EMSA assay. The NFAT binding sites [21] are indicated in red and by red boxes. (B) IL-17 probes were allowed to interact with nuclear extracts from PHA-PBMCs in an EMSA assay. Lane 1, free probes; lane 2, biotin-labeled probes plus nuclear extracts; lane 3, biotin-labeled probes plus nuclear extracts plus a 50-fold molar excess of unlabeled probes. The figure shown is representative of five independent experiments. (C) IL-17 probes were allowed to interact with recombinant NFAT proteins in an EMSA assay. Lane 1, free probes; lane 2, biotin-labeled probes plus GST-NFAT; lane 3, biotin-labeled probes plus GST-NFAT plus a 50-fold molar excess of unlabeled probes; lane 4, biotin-labeled probes plus GST-NFAT plus a 50-fold molar excess of unlabeled oligomers containing a NFAT consensus site. The figure shown is representative of five independent experiments. (D) The intensity of the bands corresponding to the DNA/protein interaction (lane 2 in **Fig. 4C**) were evaluated by densitometry to compare the binding affinity of the 197A allele and 197G allele for recombinant NFAT. The values are represented as arbitrary units (au). **indicates *P*<0.01.

To substantiate the functional relevance of the rs2275913 SNP, an EMSA assay was performed. Oligomers containing the 197A or 197G variants were biotin-labeled and allowed to interact with nuclear extracts derived from PHA-PBMCs. Despite the fact that the probes differed in just one nucleotide (A/G), the shift band corresponding to 197A probe-protein complexes was significantly more intense than that corresponding to 197G probe-protein complexes (**Fig. 4B**), thus suggesting that the two alleles have different affinities for some transcription factor in the nuclear extracts. A 50-fold excess of unlabeled IL-17 probes abrogated the formation of DNA-protein complexes, confirming the specificity of these interactions. Since NFAT has been demonstrated to play a crucial role in the regulation of IL-17 production [21] and the rs2275913 SNP maps to within the NFAT binding motif (**Fig. 4A**), DNA-protein interactions were subsequently carried out using recombinant NFAT instead of the nuclear extracts. The 197A probe-NFAT complexes displayed more a intense band than 197G probe-NFAT complexes (**Figs. 4C, D**), which were both completely eliminated by adding a competitor with a 50-fold excess of unlabeled IL-17 probes or an oligonucleotide containing a known NFAT target consensus in the binding reaction, thus suggesting that NFAT is the transcription factor which binds with differential affinities to the IL-17 probes.

## Discussion

The present study showed that the 197A allele of the IL-17 gene in the donors was associated with a higher risk of acute GVHD after unrelated fully HLA-matched BMT through the JMDP. The reason that this association did not significantly influence the TRM and OS might have resulted from the low incidence of acute GVHD-related mortality, regardless of the donor IL-17 genotype in the present cohort. Of note we have found that 197A allele positive PBMCs can produce IL-17 more efficiently than 197A allele negative PBMCs, which has not been reported so far, thus implying that the high inducibility of IL-17 might be correlated with the development of acute GVHD.

The role of IL-17 in the pathogenesis of acute GVHD remains unclear. In several mouse model experiments transfer of IL-17 producing cells induced acute GVHD [15], [16], [17], while in contrast there is a report [13] showing that donor IL-17 producing cells ameliorated acute GVHD. Host dendritic cells (DCs) are critical in the initiation of acute GVHD [39], [40], [41], thus leading to a hypothesis that IL-17 producing cells could modify the function of host DCs through unknown mechanisms. Direct interaction between IL-17 and host DCs may be supported by the fact that DCs expressed IL-17 receptors [1].

The IL-17 197A allele, which was associated with the higher production of IL-17 in comparison with the 197G allele, exhibited a higher promoter activity, as well as a higher affinity to transcriptional factor NFAT. The functional relevance of rs2275913 SNP was supported by the findings in our gene reporter assay showing that the higher promoter activity induced by 197A allele was stronger in the presence of T cell receptor activation by anti-CD3 and anti-CD28 treatment which is an upstream event in NFAT induction whereas in conditions leading to NFAT inactivation, namely CsA treatment, the differences in promoter activity induced by the 197A and 197G constructs were completely abrogated. Consistent with these observations, EMSA assay using recombinant NFAT proteins directly demonstrated a higher in affinity of 197A allele. NFAT is a transcription factor crucial for the regulation of T cell-mediated IL-17 gene transcription [21], and the rs2275913 SNP is located in the promoter adjacent to the NFAT binding region (**Fig. 4A**). These findings suggest that the rs2275913 SNP plays a functional role in the promoter activity of the IL-17 gene through influencing the transcriptional activity of NFAT, affecting the production of IL-17 from T cells.

Previous studies have reported an association between the G197A SNP in the IL-17 promoter region and the susceptibility of the Japanese population to ulcerative colitis [19], as well as to rheumatoid arthritis in the Caucasian population [18]. The present study demonstrated that the 197A genotype is related to high IL-17 production, and the results of a previous Japanese study [19] showed that the 197A genotype was a risk factor for the development of ulcerative colitis. Together, these results may explain the previous observations of increased expression of IL-17 in patients with inflammatory bowel disease such as ulcerative colitis, which promotes the recruitment of inflammatory cells into the intestinal mucosa through an increase in chemoattractants and the expression of adhesion molecules [4], [42], [43], [44]. However, another study from Norway [18] suggested an association between the 197A genotype and resistance to developing rheumatoid arthritis Since many studies have demonstrated higher levels of IL-17 in patients with rheumatoid arthritis and the essential roles of IL-17 in mediating joint damage [45], [46], [47], the G197A SNP might affect the initiation of rheumatoid arthritis, but not disease progression and severity. This issue should thus be clarified using larger cohort studies in the future.

Our earlier report [34] showed an association with the IL-17 197A genotype in the recipient, but not the donor, as in the present study, with a higher incidence of acute graft-versus-host disease. However, unlike in the previous study [34], the current cohort mainly consisted of patients receiving relatively recent transplants, including reduced-intensity transplantation. The reason for these discrepancies is unclear, because the year of the transplant and conditioning intensity were considered as co-factors in the multivariate analysis. This issue should be clarified by further investigations in patients at higher risk for acute GVHD, including those receiving peripheral blood stem cell or HLA-mismatched transplants.

In conclusion, we have reported that the G197A SNP in the IL-17 promoter predicts the development of acute GVHD and plays a functionally important role in the regulation of IL-17 production. Given that the 197A allele is significantly associated with the higher production of IL-17, G197A genotyping may be used to predict the susceptibility and severity of other IL-17-related diseases and complications including rheumatoid arthritis, periodontal disease, multiple sclerosis, allergic rhinitis, psoriasis, inflammatory bowel disease, and organ allograft rejection [11]. Furthermore, a better understanding of the molecular mechanism by which this promoter SNP controls the production of IL-17 may therefore offer some novel therapeutic insights into the mechanisms of such diseases.
